# Dr. W. H. Long

**Published:** 1884-02

**Authors:** 


### DR. W. H. LONG.

Died.—At Parsons, Kansas, December 15, 1883, of peritonitis, Dr. William Herbert Long, aged 28 years.

How truly has it been said, "Death borders upon our birth and our cradle stands in the grave," and how vividly was it brought to our mind in learning of the untimely taking away of our friend W. H. Long.

Dr. Long was born near Hagerstown, Maryland, in the year 1855. In 1874 he commenced the study of dentistry, in the office of Dr. J. E. Swallow, of Hagerstown, where he remained until 1878. Upon the completion of his studies he sought about him for a point from which to begin his life's work. In the full vigor of youth he came west, and located in the beautiful town of Parsons, Kansas, where he remained to the time of his death, enjoying from the first the confidence of its best citizens. The writer knew him well, and esteemed him as one who gave promise of much good to his chosen profession in years to come. Dignified though unassuming in his manner, and possessing a well cultivated mind, he was a most entertaining companion, and we shall always look back with profit upon the many pleasant hours we have spent with him. Placing the dignity of his profession above all other considerations, he did everything in his power to maintain it, very frequently to his pecuniary detriment. As an operator he was careful and painstaking, and leaves a record for superiority rarely enjoyed by one so young. In his premature death the dental profession of the West sustains a loss it could illy afford. The suffering during his fatal illness was excruciating to the last, but was borne with that firmness and fortitude which marks the perfect man. He leaves, besides other relatives, a faithful, loving young wife, to whom goes out the sympathy of all who knew him. Irvine.

